# Retraction: The Anticancer Potential of Chlorine Dioxide in Small-Cell Lung Cancer Cells

**DOI:** 10.7759/cureus.r194

**Published:** 2025-10-16

**Authors:** Salih Zeki Yıldız, Cemil Bilir, Gamze Guney Eskiler, Filiz Bilir

**Affiliations:** 1 Department of Chemistry, Faculty of Arts and Sciences, Sakarya University, Sakarya, TUR; 2 Medical Oncology, Istinye University, VM Medical Park Pendik Hospital, Istanbul, TUR; 3 Department of Medical Biology, Faculty of Medicine, Sakarya University, Sakarya, TUR; 4 Department of Gynecologic Oncology, Faculty of Medicine, Kocaeli University, Kocaeli, TUR

This article has been retracted by the Editors-in-Chief due to the presence of fundamental errors and methodological flaws which undermine the credibility of the study’s results and conclusions. The authors disagree with the decision to retract.

